# Dimethyl 2,7-di-*tert*-butyl­pyrene-4,9-di­carboxyl­ate

**DOI:** 10.1107/S2414314626002488

**Published:** 2026-03-11

**Authors:** Tetsuji Moriguchi, Rea Okuyama, Misa Sasaki, Noriko Miyoshi

**Affiliations:** a Department of Material Science, Faculty of Engineering, Kyushu Institute of, Technology, 1-1 Sensui-cho, Tobata-ku Kitakyushu, Fukuoka, Japan; b Technical Support Department, Management Headquarters, Kyushu Institute of, Technology, 1-1 Sensui-cho, Tobata-ku, Kitakyushu 804-8550, Japan; University of Aberdeen, United Kingdom

**Keywords:** crystal structure, polyaromatic hydro­carbon, pyrene di­carb­oxy­lic acid, di­methyl­ester

## Abstract

The complete mol­ecule of the title compound, C_28_H_30_O_4_, is generated by a crystallographic centre of symmetry and the ester moiety is twisted away from the fused-ring plane by 28.03 (8)° due to steric repulsion. In the crystal, a weak C—H⋯O hydrogen bond links the mol­ecules into (001) sheets.

## Abstract 2

The title compound crystallizes in space group *Pbca* with four mol­ecules in the unit cell.

## Graphical abstract

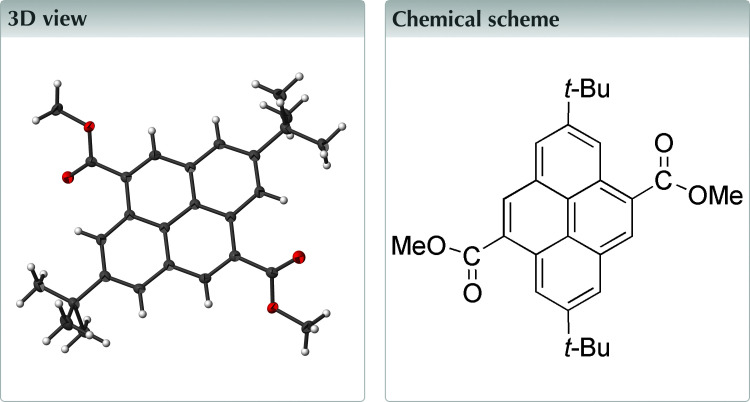

## Structure description

In recent years, polycyclic aromatic hydro­carbons (PAHs) have attracted great inter­est owing to their significant photochemical and electrical properties (Dötz *et al.*, 2000 ▸). In PAHs, pyrene is the most studied and an important class of polyaromatic hydro­carbon found in charcoal. Pyrene and its substituted derivatives have *p*-type semiconductor properties (Moriguchi *et al.*, 2017 ▸). We reported substituted pyrene derivatives (Moriguchi *et al.*, 2018 ▸) and we have also studied a lanthanide complex having four pyrene moieties (Moriguchi *et al.*, 2014 ▸) in order to evaluate its fluorescence property.

**Figure 1:**
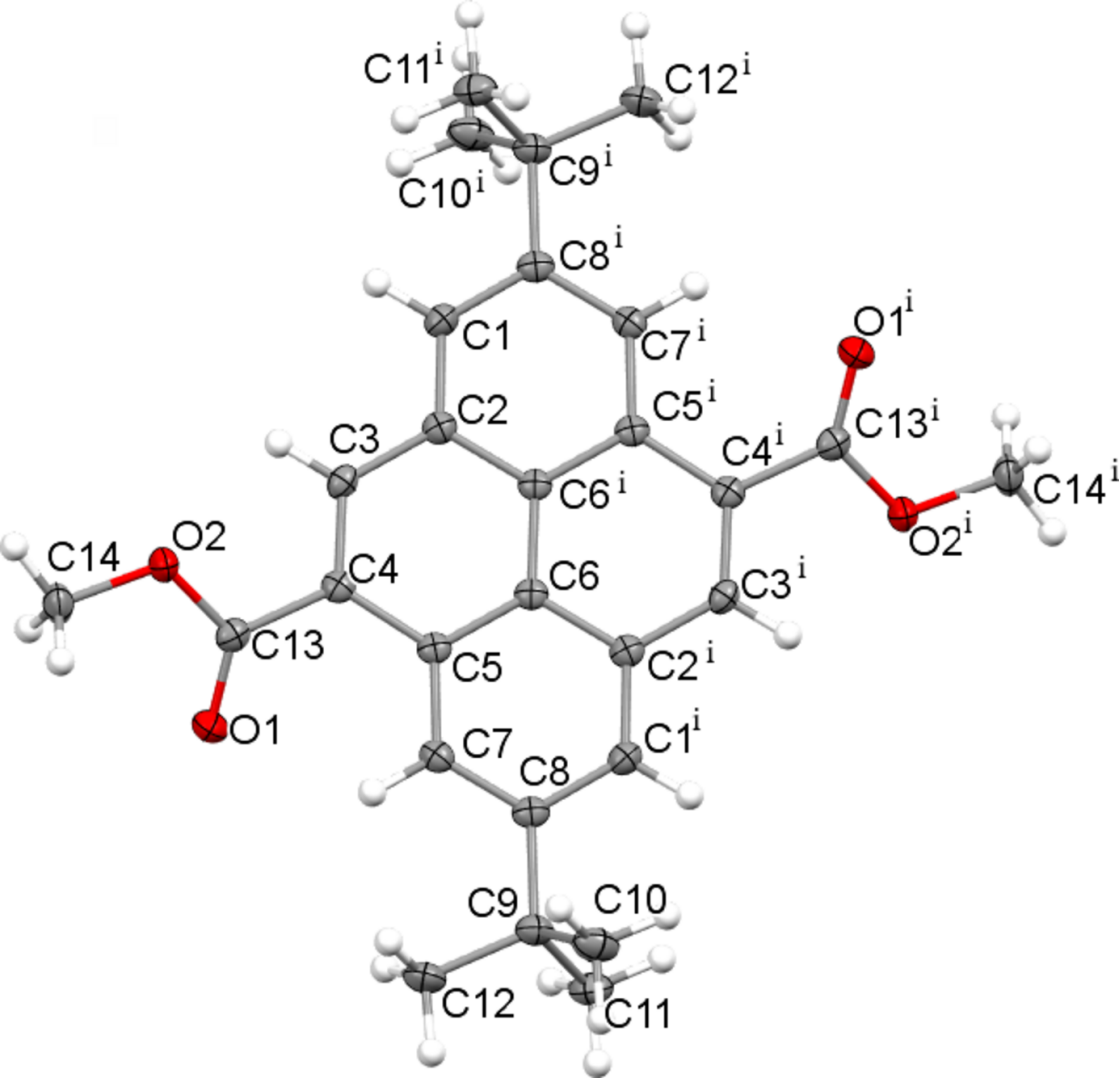
The mol­ecular structure of (**I**) with displacement ellipsoids shown at the 50% probability level. Symmetry code: (i) −*x*, 1 − *y*, 2 − *z*.

As part of our ongoing studies of these systems, we now report the synthesis and crystal structure of the title compound, C_28_H_30_O_4_ (**I**). The complete mol­ecule (Fig. 1 ▸) is generated by a crystallographic centre of symmetry in the ortho­rhom­bic space group *Pbca* at the mid-point of the C6—C6^i^ [symmetry code: (i) −*x*, 1 − *y*, 2 − *z*] bond. The C12 methyl group lies almost in the plane of the fused ring system, whereas C10 and C11 are equally displaced either side [deviations = 0.001 (2), −1.290 (2) and 1.210 (2) Å, respectively]. The twist angle between the fused ring system and the C13/O1/O2/C14 ester moiety is 28.03 (8)°. This twist appears to arise due to steric repulsion between the O atoms of the ester group and hydrogen atoms of the pyrene ring system (H7⋯O1 = 2.27 Å; H3⋯O2 = 2.36 Å).

**Figure 2:**
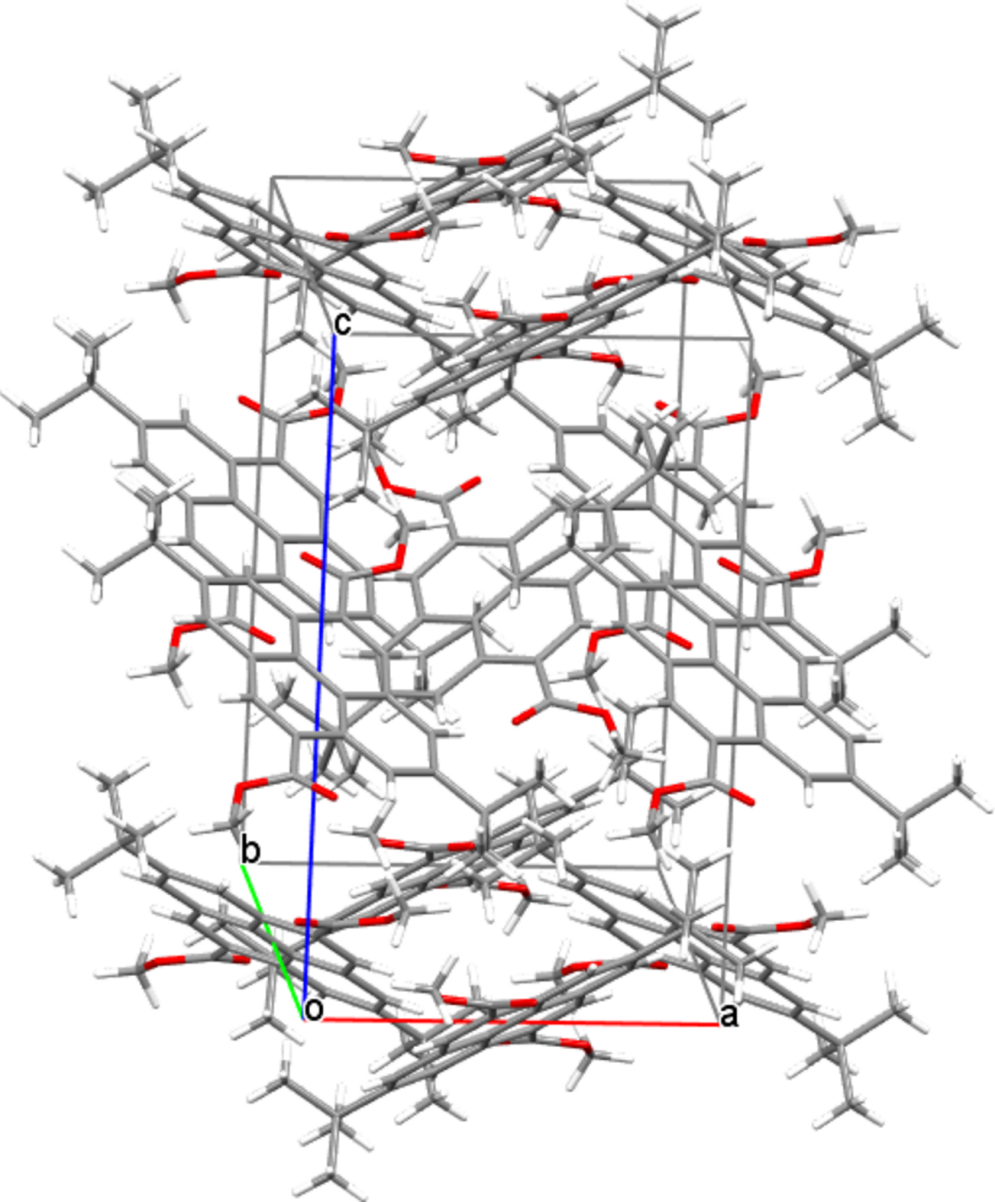
Crystal packing of (**I**).

**Figure 3:**
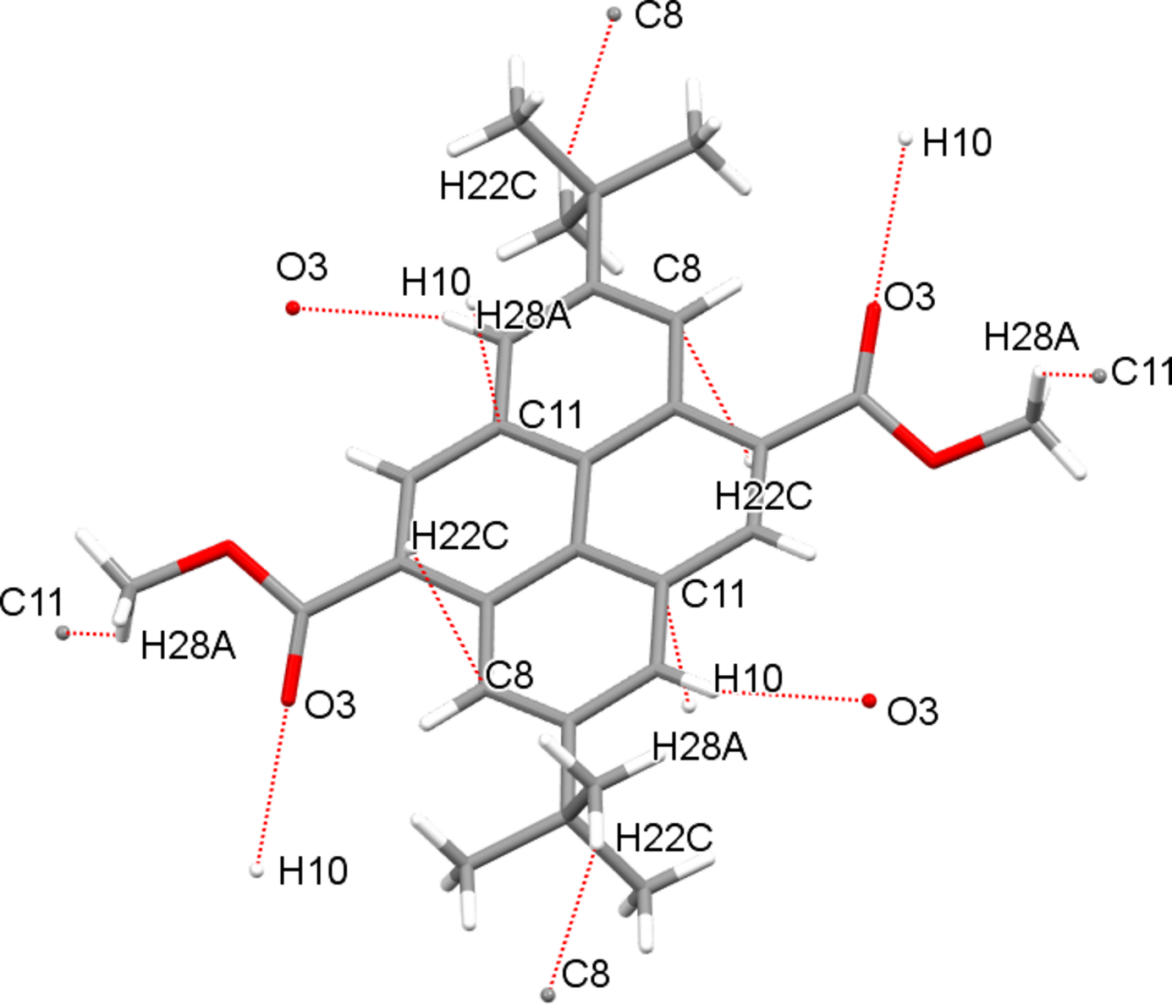
Inter­molecular short contacts in the crystal of (**I**).

The packing of (**I**) is shown in Fig. 2 ▸. No inter­molecular π–π stacking inter­actions between the pyrene rings are observed, but some short inter­molecular contacts can be detected (Fig. 3 ▸), including a weak C1—H1⋯O1^ii^ [symmetry code: (ii) = −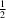 + *x*, 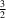 − *y*, 2 − *z*] hydrogen bond with H⋯O = 2.32 Å and C—H⋯O = 164°, which links the mol­ecules into (001) sheets.

## Synthesis and crystallization

NaOH (5.00 mmol) was added to an absolute methanol solution (50 ml) of 2,7-di-*t*-butyl­pyrene-4,9-di­carb­oxy­lic acid (1.00 mmol) at room temperature. The reaction mixture was stirred for 10 h at 318 K. After completion of reaction, the resultant mixture was cooled to room temperature, then poured into ice-cold water. The precipitate was separated by filtration and then washed with cold water. The resulting precipitate was filtered and recrystallized from methanol solution. Single crystals of (**I**) suitable for X-ray analysis were obtained by slow evaporation of di­chloro­methane solution at room temperature.

## Refinement

Crystal data, data collection and structure refinement details are summarized in Table 1 ▸.

## Supplementary Material

Crystal structure: contains datablock(s) I. DOI: 10.1107/S2414314626002488/hb4556sup1.cif

Supporting information file. DOI: 10.1107/S2414314626002488/hb4556Isup2.cml

CCDC reference: 2535992

Additional supporting information:  crystallographic information; 3D view; checkCIF report

## Figures and Tables

**Table 1:** Experimental details

Crystal data	
Chemical formula	C_28_H_30_O_4_
*M*_r_	430.52
Crystal system, space group	Orthorhombic, *Pbca*
Temperature (K)	90
*a*, *b*, *c* (Å)	9.9087 (10), 13.7094 (14), 16.7900 (17)
*V* (Å^3^)	2280.8 (4)
*Z*	4
Radiation type	Mo *K*α
μ (mm^−1^)	0.08
Crystal size (mm)	0.40 × 0.35 × 0.30

Data collection	
Diffractometer	Bruker APEXII CCD
Absorption correction	Multi-scan (*SADABS*; Krause *et al.*, 2015 ▸)
*T*_min_, *T*_max_	0.663, 0.746
No. of measured, independent and observed [*I* > 2σ(*I*)] reflections	20373, 2009, 1686
*R*_int_	0.047
(sin θ/λ)_max_ (Å^−1^)	0.595

Refinement	
*R*[*F*^2^ > 2σ(*F*^2^)], *wR*(*F*^2^), *S*	0.038, 0.092, 1.06
No. of reflections	2009
No. of parameters	149
H-atom treatment	H-atom parameters constrained
Δρ_max_, Δρ_min_ (e Å^−3^)	0.18, −0.20

Computer programs: *APEX2* and *SAINT* (Bruker, 2009 ▸), *SHELXS* (Sheldrick, 2008 ▸), *SHELXL2019/3* (Sheldrick, 2015 ▸) and *OLEX2* (Dolomanov *et al.*, 2009 ▸).

## full crystallographic data

### Dimethyl 2,7-di-tert-butylpyrene-4,9-dicarboxylate Crystal data

**Table:**

C_28_H_30_O_4_	*D*_x_ = 1.254 Mg m^−^^3^
*M**_r_* = 430.52	Mo *K*α radiation, λ = 0.71073 Å
Orthorhombic, *P**b**c**a*	Cell parameters from 4114 reflections
*a* = 9.9087 (10) Å	θ = 2.8–26.5°
*b* = 13.7094 (14) Å	µ = 0.08 mm^−^^1^
*c* = 16.7900 (17) Å	*T* = 90 K
*V* = 2280.8 (4) Å^3^	Prism, clear light yellow
*Z* = 4	0.40 × 0.35 × 0.30 mm
*F*(000) = 920	

### Dimethyl 2,7-di-tert-butylpyrene-4,9-dicarboxylate Data collection

**Table:**

Bruker APEXII CCD diffractometer	2009 independent reflections
Radiation source: sealed tube	1686 reflections with *I* > 2σ(*I*)
Graphite monochromator	*R*_int_ = 0.047
Detector resolution: 8 pixels mm^-1^	θ_max_ = 25.0°, θ_min_ = 2.8°
φ and ω scans	*h* = −11→11
Absorption correction: multi-scan (SADABS; Krause *et al.*, 2015)	*k* = −16→16
*T*_min_ = 0.663, *T*_max_ = 0.746	*l* = −19→19
20373 measured reflections	

### Dimethyl 2,7-di-tert-butylpyrene-4,9-dicarboxylate Refinement

**Table:**

Refinement on *F*^2^	Primary atom site location: structure-invariant direct methods
Least-squares matrix: full	Hydrogen site location: inferred from neighbouring sites
*R*[*F*^2^ > 2σ(*F*^2^)] = 0.038	H-atom parameters constrained
*wR*(*F*^2^) = 0.092	*w* = 1/[σ^2^(*F*_o_^2^) + (0.0411*P*)^2^ + 0.9455*P*] where *P* = (*F*_o_^2^ + 2*F*_c_^2^)/3
*S* = 1.06	(Δ/σ)_max_ = 0.001
2009 reflections	Δρ_max_ = 0.18 e Å^−^^3^
149 parameters	Δρ_min_ = −0.20 e Å^−^^3^
0 restraints	

### Dimethyl 2,7-di-tert-butylpyrene-4,9-dicarboxylate Special details

**Table:**

Geometry. All esds (except the esd in the dihedral angle between two l.s. planes) are estimated using the full covariance matrix. The cell esds are taken into account individually in the estimation of esds in distances, angles and torsion angles; correlations between esds in cell parameters are only used when they are defined by crystal symmetry. An approximate (isotropic) treatment of cell esds is used for estimating esds involving l.s. planes.

### Dimethyl 2,7-di-tert-butylpyrene-4,9-dicarboxylate Fractional atomic coordinates and isotropic or equivalent isotropic displacement parameters (Å^2^)

**Table:**

	*x*	*y*	*z*	*U*_iso_*/*U*_eq_	
O1	−0.00597 (11)	0.82315 (7)	0.89804 (6)	0.0251 (3)	
O2	−0.22549 (10)	0.78601 (7)	0.90258 (6)	0.0213 (3)	
C1	−0.27651 (14)	0.50355 (11)	1.08192 (8)	0.0182 (3)	
H1	−0.354287	0.542997	1.089500	0.022*	
C2	−0.17052 (14)	0.53904 (10)	1.03565 (8)	0.0166 (3)	
C3	−0.17815 (15)	0.63220 (10)	0.99780 (8)	0.0173 (3)	
H3	−0.257346	0.670314	1.004777	0.021*	
C4	−0.07638 (14)	0.66847 (10)	0.95215 (8)	0.0165 (3)	
C5	0.04671 (14)	0.61282 (10)	0.94065 (8)	0.0155 (3)	
C6	0.05351 (14)	0.51869 (10)	0.97619 (8)	0.0149 (3)	
C7	0.15649 (14)	0.64500 (10)	0.89476 (8)	0.0171 (3)	
H7	0.152365	0.707844	0.871049	0.021*	
C8	0.27134 (14)	0.58810 (10)	0.88272 (8)	0.0173 (3)	
C9	0.39102 (15)	0.62209 (11)	0.83199 (9)	0.0200 (3)	
C10	0.41008 (17)	0.55038 (12)	0.76256 (9)	0.0278 (4)	
H10A	0.329155	0.550616	0.729009	0.042*	
H10B	0.424908	0.484584	0.783640	0.042*	
H10C	0.488348	0.570193	0.730754	0.042*	
C11	0.51910 (15)	0.62302 (12)	0.88319 (9)	0.0262 (4)	
H11A	0.596640	0.641106	0.850099	0.039*	
H11B	0.533827	0.558011	0.905842	0.039*	
H11C	0.508712	0.670556	0.926348	0.039*	
C12	0.36998 (16)	0.72414 (11)	0.79755 (10)	0.0262 (4)	
H12A	0.289826	0.724224	0.763359	0.039*	
H12B	0.449211	0.742784	0.766115	0.039*	
H12C	0.357403	0.770847	0.841110	0.039*	
C13	−0.09487 (14)	0.76657 (10)	0.91529 (8)	0.0178 (3)	
C14	−0.25447 (16)	0.88024 (11)	0.86837 (9)	0.0252 (4)	
H14A	−0.223178	0.931577	0.904554	0.038*	
H14B	−0.351987	0.886744	0.860213	0.038*	
H14C	−0.207928	0.886423	0.817121	0.038*	

### Dimethyl 2,7-di-tert-butylpyrene-4,9-dicarboxylate Atomic displacement parameters (Å^2^)

**Table:**

	*U*^11^	*U*^22^	*U*^33^	*U*^12^	*U*^13^	*U*^23^
O1	0.0216 (6)	0.0183 (6)	0.0356 (6)	−0.0033 (5)	0.0012 (5)	0.0032 (5)
O2	0.0196 (6)	0.0175 (5)	0.0268 (6)	0.0018 (4)	−0.0005 (4)	0.0051 (4)
C1	0.0155 (8)	0.0204 (8)	0.0189 (7)	0.0011 (6)	0.0002 (6)	−0.0021 (6)
C2	0.0165 (7)	0.0187 (8)	0.0145 (7)	−0.0011 (6)	−0.0019 (6)	−0.0022 (6)
C3	0.0157 (7)	0.0192 (8)	0.0169 (7)	0.0020 (6)	−0.0014 (6)	−0.0029 (6)
C4	0.0168 (7)	0.0174 (8)	0.0152 (7)	−0.0016 (6)	−0.0027 (6)	−0.0024 (6)
C5	0.0153 (7)	0.0178 (7)	0.0133 (7)	−0.0022 (6)	−0.0027 (6)	−0.0027 (6)
C6	0.0152 (7)	0.0170 (7)	0.0126 (7)	−0.0022 (6)	−0.0028 (5)	−0.0023 (5)
C7	0.0185 (8)	0.0177 (7)	0.0152 (7)	−0.0028 (6)	−0.0029 (6)	0.0006 (6)
C8	0.0164 (7)	0.0212 (8)	0.0143 (7)	−0.0032 (6)	−0.0012 (6)	−0.0025 (6)
C9	0.0185 (8)	0.0222 (8)	0.0194 (7)	−0.0033 (6)	0.0030 (6)	−0.0007 (6)
C10	0.0302 (9)	0.0299 (9)	0.0233 (8)	−0.0065 (7)	0.0085 (7)	−0.0022 (7)
C11	0.0185 (8)	0.0330 (9)	0.0270 (8)	−0.0046 (7)	0.0017 (6)	0.0014 (7)
C12	0.0246 (9)	0.0268 (9)	0.0273 (9)	−0.0044 (7)	0.0068 (7)	0.0053 (7)
C13	0.0176 (8)	0.0195 (8)	0.0164 (7)	−0.0003 (6)	0.0002 (6)	−0.0039 (6)
C14	0.0291 (9)	0.0189 (8)	0.0277 (8)	0.0067 (7)	0.0018 (7)	0.0040 (6)

### Dimethyl 2,7-di-tert-butylpyrene-4,9-dicarboxylate Geometric parameters (Å, º)

**Table:**

O1—C13	1.2088 (17)	C8—C9	1.533 (2)
O2—C13	1.3386 (17)	C9—C10	1.537 (2)
O2—C14	1.4426 (17)	C9—C11	1.533 (2)
C1—H1	0.9500	C9—C12	1.528 (2)
C1—C2	1.394 (2)	C10—H10A	0.9800
C1—C8^i^	1.391 (2)	C10—H10B	0.9800
C2—C3	1.428 (2)	C10—H10C	0.9800
C2—C6^i^	1.418 (2)	C11—H11A	0.9800
C3—H3	0.9500	C11—H11B	0.9800
C3—C4	1.361 (2)	C11—H11C	0.9800
C4—C5	1.452 (2)	C12—H12A	0.9800
C4—C13	1.492 (2)	C12—H12B	0.9800
C5—C6	1.423 (2)	C12—H12C	0.9800
C5—C7	1.404 (2)	C14—H14A	0.9800
C6—C6^i^	1.424 (3)	C14—H14B	0.9800
C7—H7	0.9500	C14—H14C	0.9800
C7—C8	1.394 (2)		
			
C13—O2—C14	115.73 (11)	C12—C9—C10	108.40 (12)
C2—C1—H1	119.1	C12—C9—C11	108.51 (12)
C8^i^—C1—H1	119.1	C9—C10—H10A	109.5
C8^i^—C1—C2	121.70 (13)	C9—C10—H10B	109.5
C1—C2—C3	121.34 (13)	C9—C10—H10C	109.5
C1—C2—C6^i^	119.96 (13)	H10A—C10—H10B	109.5
C6^i^—C2—C3	118.69 (13)	H10A—C10—H10C	109.5
C2—C3—H3	118.7	H10B—C10—H10C	109.5
C4—C3—C2	122.55 (13)	C9—C11—H11A	109.5
C4—C3—H3	118.7	C9—C11—H11B	109.5
C3—C4—C5	120.37 (13)	C9—C11—H11C	109.5
C3—C4—C13	118.17 (13)	H11A—C11—H11B	109.5
C5—C4—C13	121.46 (12)	H11A—C11—H11C	109.5
C6—C5—C4	117.42 (12)	H11B—C11—H11C	109.5
C7—C5—C4	123.98 (13)	C9—C12—H12A	109.5
C7—C5—C6	118.57 (13)	C9—C12—H12B	109.5
C2^i^—C6—C5	119.08 (13)	C9—C12—H12C	109.5
C2^i^—C6—C6^i^	119.14 (16)	H12A—C12—H12B	109.5
C5—C6—C6^i^	121.78 (16)	H12A—C12—H12C	109.5
C5—C7—H7	118.8	H12B—C12—H12C	109.5
C8—C7—C5	122.42 (13)	O1—C13—O2	122.59 (13)
C8—C7—H7	118.8	O1—C13—C4	126.04 (13)
C1^i^—C8—C7	118.27 (13)	O2—C13—C4	111.37 (12)
C1^i^—C8—C9	118.91 (13)	O2—C14—H14A	109.5
C7—C8—C9	122.82 (13)	O2—C14—H14B	109.5
C8—C9—C10	108.80 (12)	O2—C14—H14C	109.5
C8—C9—C11	109.36 (12)	H14A—C14—H14B	109.5
C11—C9—C10	109.21 (13)	H14A—C14—H14C	109.5
C12—C9—C8	112.52 (12)	H14B—C14—H14C	109.5
			
C1—C2—C3—C4	−179.81 (13)	C5—C7—C8—C1^i^	−0.3 (2)
C1^i^—C8—C9—C10	59.99 (17)	C5—C7—C8—C9	179.30 (12)
C1^i^—C8—C9—C11	−59.22 (17)	C6^i^—C2—C3—C4	−1.3 (2)
C1^i^—C8—C9—C12	−179.88 (13)	C6—C5—C7—C8	0.1 (2)
C2—C3—C4—C5	−0.6 (2)	C7—C5—C6—C2^i^	0.20 (19)
C2—C3—C4—C13	179.95 (12)	C7—C5—C6—C6^i^	179.69 (15)
C3—C4—C5—C6	2.36 (19)	C7—C8—C9—C10	−119.65 (15)
C3—C4—C5—C7	−179.79 (13)	C7—C8—C9—C11	121.14 (15)
C3—C4—C13—O1	152.38 (14)	C7—C8—C9—C12	0.48 (19)
C3—C4—C13—O2	−27.83 (18)	C8^i^—C1—C2—C3	178.31 (13)
C4—C5—C6—C2^i^	178.17 (12)	C8^i^—C1—C2—C6^i^	−0.1 (2)
C4—C5—C6—C6^i^	−2.3 (2)	C13—C4—C5—C6	−178.16 (12)
C4—C5—C7—C8	−177.69 (13)	C13—C4—C5—C7	−0.3 (2)
C5—C4—C13—O1	−27.1 (2)	C14—O2—C13—O1	−1.3 (2)
C5—C4—C13—O2	152.68 (12)	C14—O2—C13—C4	178.87 (11)

Symmetry code: (i) −*x*, −*y*+1, −*z*+2.

### Dimethyl 2,7-di-tert-butylpyrene-4,9-dicarboxylate Hydrogen-bond geometry (Å, º)

**Table:**

*D*—H···*A*	*D*—H	H···*A*	*D*···*A*	*D*—H···*A*
C1—H1···O1^ii^	0.95	2.38	3.3057 (18)	164

Symmetry code: (ii) *x*−1/2, −*y*+3/2, −*z*+2.
